# The PD-1, PD-L1 expression and CD3+ T cell infiltration in relation to outcome in advanced gastric signet-ring cell carcinoma, representing a potential biomarker for immunotherapy

**DOI:** 10.18632/oncotarget.16407

**Published:** 2017-03-21

**Authors:** Shenying Jin, Bo Xu, Lixia Yu, Yao Fu, Hongyan Wu, Xiangshan Fan, Jia Wei, Baorui Liu

**Affiliations:** ^1^ The Comprehensive Cancer Centre of Drum Tower Hospital, Medical School of Nanjing University & Clinical Cancer Institute of Nanjing University, Nanjing 210008, China; ^2^ The Comprehensive Cancer Center of Drum Tower Hospital, Clinical College of Nanjing Medical University, Nanjing 210008, China; ^3^ Department of Pathology, Nanjing Drum Tower Hospital, The Affiliated Hospital of Nanjing University Medical School, Nanjing 210008, China

**Keywords:** programmed cell death protein 1, programmed cell death ligand 1, CD3+ T cell, microsatellite instability, gastric signet-ring cell carcinoma

## Abstract

Recent data supports a potentially significant role for immune checkpoint inhibitors in the treatment of gastric cancer. However, there are few data on the clinical implications of immunotherapy markers in gastric signet-ring cell carcinoma (SRCC). We evaluated the expression of programmed cell death protein-1 (PD-1), programmed cell death ligand 1(PD-L1), infiltration by CD3+ T cell, microsatellite instability (MSI), and Epstein-Barr Virus (EBV), and the relationship of each factor to survival in 89 advanced SRCC patients. All patients received 5-FU-based first-line chemotherapy. PD-L1 and PD-1 were expressed in 40.4% and 18.0% of the patients, respectively. There was a significant correlation between PD-L1 and PD-1 expression (r=0.363, p<0.001). There was loss of at least 1 of the 4 DNA mismatch repair (DNA-MMR) gene proteins in 32.6% of samples. Only 1 case out of 89 was EBV positive, with concurrent PD-L1 positivity, a high degree of CD3+ T cell infiltration and MSI. Increased CD3+ T cells numbers was associated with increased PD-1 expression (r=0.256, p=0.012) and MSI status (r=0.208, p=0.049). High CD3+ T cell infiltration was related to better OS (23.7 months, 95% CI: 19.0-38.0 *vs* 15.8 months, 95% CI: 13.0-22.0, p=0.033), but was not an independent prognostic factor for survival after multivariate analysis (HR=0.68, 95% CI: 0.42-1.10, p=0.116). CD3+ T cell was more infiltrated in PD-1 positive, tumors with MSI and were associated with better OS, indicating an adaptive immune resistance may be occurring. Further research into the cancer immunotherapy markers of SRCC immune microenvironment may highlight targets for immunotherapy.

## INTRODUCTION

Gastric cancer (GC) consists 8% of general tumor cases and 10% of tumor-associated deaths worldwide, having an estimated 989,600 new occurred incidence and 738,000 deaths annually. Approximately 40% of GC in China, with patients commonly presenting with advanced disease [1]. The median survival time for those with advanced GC is less than 1 year [2]. Chemotherapy remains the major treatment for advanced GC. Thus far, only Her-2 and VEGF have been approved as targets for molecular pathways in GC. Chemotherapy in combination with targeted therapies, including trastuzumab [3] and ramucirumab [4], has resulted in modest improvements in survival despite not providing a long-term cure. An estimated 9.1% of patients with gastric carcinoma were Signet ring cell carcinoma (SRCC) [5]. Advanced gastric SRCC is generally considered to be more invasive, has greater likelihood of lymph node metastasis than other cell types, and has a worse prognosis and is less sensitive to chemotherapy than non-SRCC [6–8]. Furthermore, SRCC can't benefit a lot from target therapy because a low expression level of Her-2. It is still uncertain whether SRCC patients can profit from a specific therapy.

Among recent years, immunotherapeutic agents targeting immunosuppressive proteins have attracted more and more attention in cancer therapy, especially those targeting the PD-1 pathway [9]. Immune checkpoint inhibitors such as anti-PD-1/anti-PD-L1 show the effects in inducing long-term regression and prolonging stabilization in several kinds of tumors [10–12]. Several clinical researches of anti-PD-1/anti-PD-L1 are carrying on in patients with advanced GC, and some have revealed impressive tumor response to immune checkpoint inhibitors [13–15]. In fact, only a small part of patients can benefit from anti-PD-1, so convincing markers are needed for guiding the use of PD-1 inhibitors. Questions still remain that which subgroup of GC will benefit from the treatment of checkpoint inhibitors.

Tumor-infiltrating lymphocytes (TILs) have been detected among several kinds of tumors, thought to be a convincing prognostic biomarker in cancer [16–18]. Immune cells around tumor cells are considered to be an immune response against tumor, leading to a better prognosis. However, immunosuppressive proteins, including PD-L1, are also present in tumors and protect the tumor from immune attack. The interaction between tumor immunosuppressive proteins and TILs in the tumor microenvironment is still unclearly in GC.

Using data from The Cancer Genome Atlas (TCGA) [19], a recent comprehensive molecular analysis of 295GC proposed a molecular classification of GC into four subgroups: Epstein-Barr Virus (EBV) positive GC, microsatellite instability (MSI), chromosomal instability (CIN) and genomically stable (GS) GC.EBV-positive (EBV+) GC and microsatellite unstable GC tumors are the two subgroups which received most attention.

EBV+ GCs have thought to be a special subgroup with distinct clinicopathologic characteristics and better prognosis [20], results ascribed to an EBV-directed immune response that is made prominently of CD8+ cytotoxic T-cell infiltration [21]. Suppressing immune response through activation PD-1 pathways may be crucial for EBV+GC. Therefore, PD-L1 and PD-L2 may protect EBV+ GCs from the host immune surveillance.

MSI tumors have attracted much attention on account of favorable outcomes compared with those MSS tumors [22]. MSI tumors have revealed a distinct advantange over microsatellite stable (MSS) tumors in response to anti-PD-1 immunotherapy [23].

The immune response seems to be important in the progression of GC. However, few studies have evaluated the prognostic value of PD-L1, PD-1, CD3+T cells, MSI, and EBV in SRCC patients.

Given the therapeutic potential of PD-L1/PD-1-based therapies, we sought to evaluate PD-L1 and PD-1 expression in SRCC. We examined the CD3+ TILs infiltration in the tumor microenvironment, as well as EBV infection and MSI phenotype. Finally, the prognostic values of PD-L1, PD-1, CD3+ TILs, and MSI phenotype were evaluated.

## RESULTS

### Patient characteristics

Of the 89 patients sampled, 75.3% of the patients were male. The median age was 57 years (range, 32-81 years). At the time of initial diagnosis, 80 (89.9%) were classified as stage III-13 (13.5%) were stage IIIA, 47 (52.8%) were stage IIIB, 21 (23.6%) were stage IIIC. Nine patients (10.1%) were classified as stage IV. Patients at stage III were all administrated first-line 5-FU-based adjuvant chemotherapy after D2 gastrectomy, while patients at stage IV were treated by first-line 5-FU-based palliative chemotherapy. Overall survival data was available in 86 (96.6%) cases. The mean follow-up period was 28.0 months (range, 3.0 -135.4 months). To our best knowledge, there is no research on the relationship of biomarkers and chemotherapy efficacy. Therefore, in the present study, we didn't estimate correlation between markers and chemotherapy efficacy.

### Expression of PD-L1 and PD-1 in SRCC

**Figure 1:**
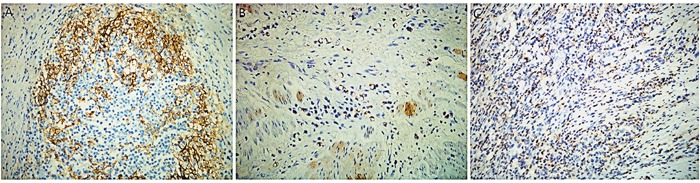
Immunohistochemical staining for programmed death-1 ligand-1 (PD-L1) and programmed death-1 (PD-1) in gastric signet-ring cell carcinoma (SRCC) tissue **(A)** Representative immunohistochemical staining for PD-1 cells infiltrating gastric cancer tissue. **(B)** Representative immunohistochemical staining for PD-L1 in tumor cells. **(C)** Representative immunohistochemical staining for PD-L1 in immune cells. Original magnification ×400.

The expression levels of PD-L1 and PD-1 were evaluated by immunohistochemical staining in all 89 samples. The staining of PD-L1 and PD-1 was detected in membrane and cytoplasm of cells. PD-L1-positive staining was visible in tumor, stromal, and immune cells, but not in non-neoplastic gastric epithelium, while PD-1-positive staining was seen in TILs (Figure 1). Of these 89 samples, 36 (40.4%) stained positive for PD-L1. The percentage of stained tumor cells and immune cells ranged from 0-80%. PD-1-positive stroma cells were observed in 16 of 89 cases (18.0%).

PD-1 expression in TILs was significantly correlated with PD-L1 expression (p<0.000, Table 1). There was a general tendency for PD-L1 and PD-1 in SRCC to be either both or both negative positive.

**Table 1:** Correlation between PD-L1, PD-1 expression, CD3-positive TILs infiltration and MSI phenotype in SRCC patients

Spearman correlation analysis	PD-L1	PD-1	CD3	MSI
**PD-L1**	1.000	0.363(p=0.000)	0.174(p=0.103)	0.111(p=0.301)
**PD-1**	0.363(p=0.000)	1.000	0.256(p=0.012)	0.199(p=0.061)
**CD3**	0.174(p=0.103)	0.256(p=0.012)	1.000	0.208(p=0.049)
**MSI**	0.111(p=0.301)	0.199(p=0.061)	0.208(p=0.049)	1.000

A: P values were calculated using a two-sided log-rank test.

B: Abbreviations: SRCC, signet-ring cell carcinoma; PD-1, programmed cell death protein-1; PD-L1, programmed cell death ligand 1; MSI, microsatellite instability; TILs, tumor-infiltrating lymphocytes.

### CD3+ TILs infiltration in SRCC and association with PD-L1, PD-1 expression

**Figure 2:**
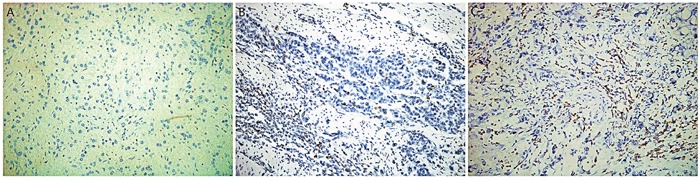
Immunohistochemical staining for CD3+ TILs infiltration in SRCC tissue **(A)** Rare CD3+ TILs infiltrated in SRCC tissue. **(B)** Representative immunohistochemical staining for low-CD3 group. **(C)** Representative immunohistochemical staining for high-CD3 group. Original magnification ×400.

**Figure 3:**
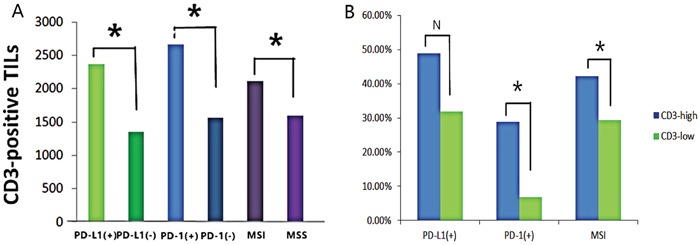
CD3+ TILs increased with the expression of PD-L1, PD-1 and MSI status **(A)** The average CD3+ TILs number with or without PD-L1/PD-1 expression and in MSI or MSS status were determined using independent-sample t-tests. CD3+ TILs were more infiltrated in PD-L1-positive (p=0.007), PD-1-positive (p=0.023), and tumor with MSI (p=0.049). **(B)** Correlation between CD3 number and PD-L1, PD-1 expression, and MSI status were determined using the Chi-square exact test. PD-L1 expression (p=0.101), PD-1 expression (p=0.072) and MSI status (p=0.049) increased with increasing CD3 number in SRCC tissue.

The number of CD3+ TILs was counted in 30 random high-power fields in representative intratumoral regions of each section, ranged from 20 to 7,915, with a median value of 1,185 (Figure 2). We next compared the average number of CD3+ cells in SRCC with and without expression of PD-L1 and PD-1. As shown in Figure 3A, the average number of CD3+ TILs was 1.8-fold higher within PD-L1-positive tumors (p=0.007) and 1.7-fold higher within PD-1-positive tumors (p=0.023). The median number of CD3+ TILs infiltrates, was used to define 'low' versus 'high' expression. The correlation between CD3+ TILs and PD-L1, PD-1 expression was then determined. An increasing number of CD3+ TILs was associated with increasing PD-1 expression in tumors (p=0.012, Table 1), while there is no association between CD3+ TILs and the expression of PD-L1 (p=0.103, Table 1). In tumors that were considered highly CD3+, 28.9% were PD-1 positive and 48.9% were PD-L1 positive. Both percentages were lower in low CD3+ tumors (Figure 3B).

### EBV infection in SRCC

**Figure 4:**
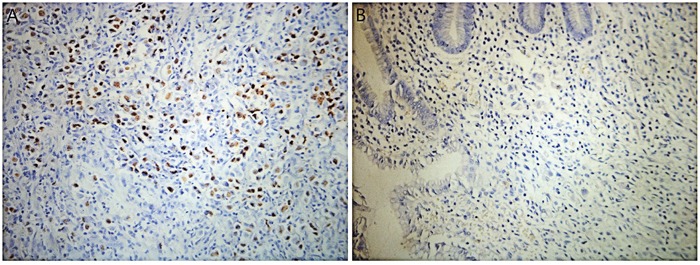
Chromogenic *in-situ* hybridization for Epstein-Barr Virus-encoded RNA (EBER) in SRCC tissue **(A)** EBER-positive highlights carcinoma cells. **(B)** EBER-negative. Original magnification ×400.

EBER-positive staining was visible in the nucleus of tumor cells. There was only 1 EBER-positive specimen in the entire cohort (Figure 4). The only EBV-associated SRCC in our study belonged to a 54-years-old male, whose surgical specimen showed membranous PD-L1 staining on tumor and immune cell and abundant CD3+ TILs in the tumor. The patient had a decreased MLH1 immunostain compared to the internal positive control, demonstrating a case of a tumor with MSI.

### MSI phenotype and SRCC

**Figure 5:**
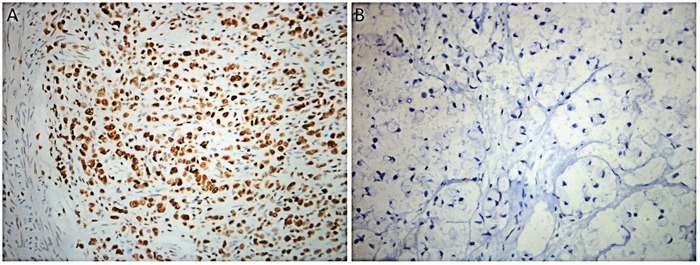
Immunohistochemical staining for MLH1, MSH2, MSH6 and PMS2 in SRCC tissue **(A)** Representative immunohistochemical staining for MLH1 in tumor cells. **(B)** Missing immunohistochemical staining of MLH1 in tumor cells. Original magnification ×400.

MSI was assessed by immunohistochemistry using antibodies directed against MLH1, PMS2, MSH2, and MSH6. Compared with the internal positive control, a total of 29 SRCC (32.6%) samples had a decreased, missing, or not evaluable immunostain of any of the 4 DNA-MMR proteins. Among the 29 samples, there was a loss of MLH1 and/or PMS2 in 25 (86.2%) cases, and 8 (27.6%) cases had complete loss of expression of MSH2 and/or MSH6 (Figure 5). Compared with patients who had MSS tumors, those with MSI were observed to have more CD3+ TILs. As shown in Figure 3A, the average number of CD3+ TILs was 1.3-fold higher within MSI tumors than MSS tumors (p=0.049, Figure 3). MSI tumors comprised 42.2% (19/45) of the tumors in high-CD3+TILs microenvironments and 29.4% (13/44) of the tumors in low-CD3+ TILs microenvironments (p=0.049), suggesting that increased CD3+ TILs numbers were associated with tumor MSI status.

### Correlations between PD-L1, PD-1 expression, CD3+ T cells, MSI, EBV, and clinicopathological factors

When analyzing the correlation between the expression of PD-L1, PD-1 and clinicopathological factors, only age was significantly related with increased PD-L1 expression. The PD-L1-positive tumors were more commonly seen in younger age patients (p=0.049; Table 2). No significant relation were found between PD-L1, PD-1 expression and gender or stage (p=1.000, p=0.646, respectively; Table 2). In tumor immune environment, the density of CD3+ TILs did not differ according to gender, age, or tumor stage. Similarly, MSI phenotype showed no association with sex, age, or tumor stage (Table 2).

**Table 2:** Associations between clinicopathologic findings and the expression of PD-1, PD-L1 in tumor, the number of immune cells in the tumor microenvironment and MSI phenotype in SRCC patients

Characteristics	PD-L1			PD-1			CD3			MSI phenotype			EBV		
	PD-L1(+)	PD-L1(−)	P*	PD-1(+)	PD-1(−)	P*	CD3-high	CD3-low	P*	MSI	MSS	P*	EBER(+)	EBER(−)	P*
**Gender**			1			0.21			0.462			0.93			
Male	27(30.3%)	40(44.9%)		10(11.2%)	57(64.0%)		32(36.0%)	35(39.3%)		22(24.7%)	45(50.6%)		1(1.1%)	66(71.2%)	
Female	9(9.1%)	13(14.6%)		6(6.7%)	16(18.0%)		13(14.6%)	9(10.1%)		7(7.9%)	15(16.9%)		0(0%)	22(24.7%)	
**Age**			0.049			0.095			0.139			0.133			
<57	12(13.5%)	30(33.7%)		11(12.4%)	31(34.8%)		25(28.1%)	17(19.1%)		17(19.1%)	25(28.1%)		1(1.1%)	41(46.1%)	
>=57	24(27.0%)	23(25.8%)		5(5.6%)	42(47.2%)		20(22.5%)	27(30.3%)		12(13.5%)	35(39.3%)		0(0%)	47(52.8%)	
**Stage**			0.646			0.627			0.073			0.484			
III	33(37.1%)	47(52.8%)		14(15.7%)	66(74.2%)		43(48.3%)	37(41.6%)		27(30.3%)	53(60.0%)		1(1.1%)	80 (89.9%)	
IV	3(3.4%)	6(6.7%)		1(1.1%)	8(9.0%)		2(2.2%)	7(7.9%)		2(2.2%)	7(7.9%)		0(0%)	9 (10.1%)	

A: P values were calculated using a two-sided log-rank test.

B: * Pearson's chi-square test and Fisher's exact test.

**C: Abbreviations:** SRCC, signet-ring cell carcinoma; PD-1, programmed cell death protein-1; PD-L1, programmed cell death ligand 1; MSI, microsatellite instability; EBV, Epstein-Barr Virus

### Prognostic significance

Follow up data was collected in 86 SRCC patients. The median OS was 19.9 months (95%CI: 16.0-23.7 months) in all 86 patients. When OS was separated by cancer stage, the OS was 21.0 months (95%CI: 17.5-26.5 months) for those at stage III and 4.2 months (95%CI: 3.0-19.8 months) for patients at stage IV (Table 3).

**Table 3:** Median overall survival and HR for risk of mortality in 86 advanced SRCC patients

Variable	Overall survivalMonths (95% CI)	P-value^*^	Risk of mortalityHR (95% CI)	P-value^^^
**Gender**		0.508		0.489
Male	19.9(16.9-26.0)		0.82(0.47-1.44)	
Female	18.7(10.0-43.5)		1.00(Reference)	
**Age**		0.551		0.178
<57	18.5(14.0-24.8)		1.00(Reference)	
>=57	20.3(15.0-27.2)		0.69(0.40-1.12)	
**TNM staging**		<0.001		<0.001
IV	21.0(17.5-26.5)		1.00(Reference)	
III	4.2(3.0-13.7)		5.20(2.38-11.32)	
**PD-L1**		0.516		0.658
Positive	21.0(18.0-32.6)		0.88(0.51-1.52)	
Negative	17.5(13.5-24.8)		1.00(Reference)	
**PD-1**		0.343		0.407
Positive	23.8(16.5-43.3)		0.76(0.39-1.46)	
Negative	18.5(15.0-22.9)		1.00(Reference)	
**CD3+TILs**		0.033		0.116
High	23.7(19.0-38.0)		0.68(0.42-1.10)	
Low	15.8(13.0-22.0)		1.00(Reference)	
**MSI status**		0.349		0.584
MSI	21.0(18.5-27.1)		1.00(Reference)	
MSS	18.0(14.2-24.4)		1.16(0.69-1.94)	
**EBV**		——		——
Positive	21		——	
Negative	19.8(16-23.7)		——	

A: P values were calculated using a two-sided log-rank test.

B: * Kaplan–Meier method.

C: ^ Cox proportional hazards model.

D: Abbreviations: SRCC, signet-ring cell carcinoma; PD-1, programmed cell death protein-1; PD-L1, programmed cell death ligand 1; MSI, microsatellite instability; EBV, Epstein-Barr Virus; CI, confidence intervals; HR, hazard ratios.

**Figure 6:**
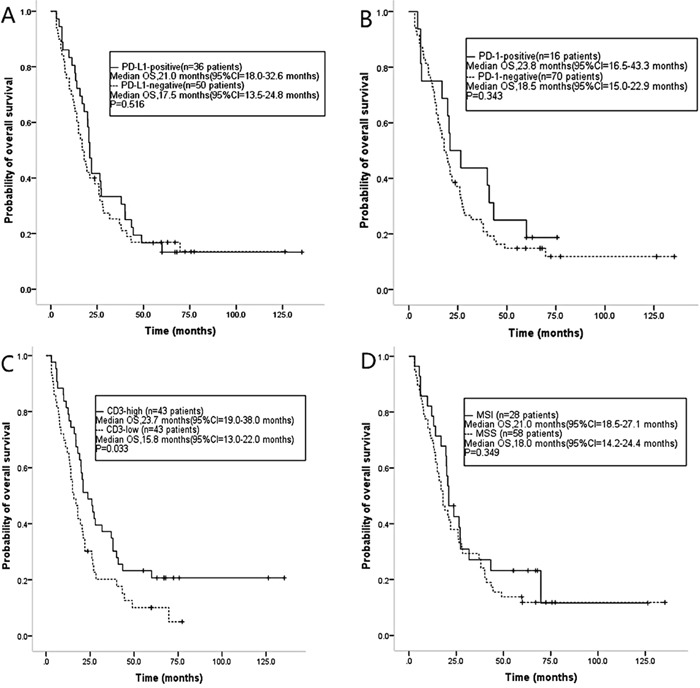
Kaplan–Meier estimates of overall survival in SRCC patients according to the expression of **(A)** PD-L1 and **(B)** PD-1, **(C)** CD3+ T cell infiltration, and **(D)** MSI status.

The median OS was 21.0 months (95% CI: 18.0-32.6 months) in PD-L1-positive cases and 17.5 months in PD-L1-negative cases (95% CI: 13.5-24.8 months). The expression of PD-L1 was related with better OS in SRCC patients, but did not have significant difference (p=0.516) (Figure 6A). Similarly, patients with PD-1-positive tumors tended to have better OS (23.8 months [95%CI: 16.5-43.3 months]) compared to those with PD-1-negative tumors (18.5 months [95%CI: 15.0-22.9 months], p=0.343, Figure 6B), although these findings were also not statistically significant. OS was also evaluated according to the number of CD3+ TILs. Median survival was 23.7 months (95%CI: 19.0-38.0 months) for those in a high-CD3+ microenvironment, compared with 15.8 months (95%CI: 13-22 months) for those in a low-CD3+ microenvironment (p=0.033, Figure 6C). No significantly difference was seen between MSI tumors and MSS tumors in terms of OS (21.0 months [95% CI: 18.5-27.1 months] vs. 18.0 months [95% CI: 14.2-24.4 months], respectively; p=0.349, Figure 6D).

To test the independent prognostic effect of these clinical factors, Cox proportional hazard models were applied, adjusting for age, gender, PD-1 and PD-1 expression, CD3+ TILs infiltration, and MSI phenotype. As shown in Table 3, only tumor stage was an independent prognostic predictor in our study.

## DISCUSSION

In European Society for Medical Oncology annual meeting of 2014, the anti-PD-L1 antibody, pembrolizumab, attracted lots of attention because of its safety and efficacy in treatment for GC [13]. It has been reported 22–27% of patients with PD-L1-expressing benefited from pembrolizumab, while only 12% of PD-L1 negative cases were response to anti-PD-1 therapy [24, 25]. Similarly, in other tumor types like malignant melanoma [26], non-small cell lung cancer [27], and urothelial bladder cancer [28], it has been shown that PD-L1-positive tumors have a significantly higher response rate to anti-PD-1/PD-L1 therapy. Treatment-independent effects on patient survival have to be considered in PD-L1/PD-1-positive GCs and have to be distinguished from therapeutic effects. PD-L1/PD-1 expression might not only serve as a prognostic biomarker, but may also be a predictive factor for response to PD-1/PD-L1 checkpoint inhibitor treatment.

In present study, PD-L1 and PD-1 expressed in 40.4% and 18.0 % of the cases (PD-L1 and PD-1 out-off value: >1%), respectively. This rate of PD-L1 positivity is similar to that found in a report informed in the American Society of Clinical Oncology of 2015, in which PD-L1 was detected in 40% of advanced GC received the treatment of the PD-1 monoclonal antibody, pembrolizumab (PD-L1 out-off value: >1%) [13]. PD-L1-positive tumors had better OS than PD-L1-negative; however, the estimates were not statistically significant. In GC, the prognostic value of PD-L1 is still debatable. In some studies, PD-L1 expression in GC was associated with favorable outcome (PD-L1 out-off value: >10%) [29], while in others, PD-L1 expression and more CD8+ T cell infiltration were related to poorer progression-free survival (PFS) and OS in GC(PD-L1 out-off value: >5% in tumor cell, >1% in immune cell) [30]. The reasons why different researches drawn these discrepant conclusions may be the small number of cases enrolled, the various kinds of monoclonal antibody and cut-off values of PD-1 and PD-L1 expression, and the discrepant samples used (frozen or FFPE tumor tissue). Several questions remain with respect to the prognostic value of PD-L1 in SRCC patients: How many cases need to be examined in research to robustly contribute to the literature? What needs to be considered in the development of a PD-L1 biomarker score? What is the appropriate cut-off value to discern the level of expression?

In breast cancer, it has been reported that the PD-L1 expression was closely related to the number of infiltrated regulatory T-cells (T-regs). Therefore, PD-L1 and T-regs build a suppressive immune microenvironment [31]. In melanoma, PD-1-positive TILs have showed to suppress T cell activation and impair T cell function [32]. In addition, the expression of PD-L1 also contributes to the epithelial-to-mesenchymal transition (EMT) of tumor cells [33]. Collectively, the above evidences support that upregulating PD-1 expression levels may help tumors to evade immune attack and develop. Further exploration into the interaction of PD-L1 and prognosis in GC is urgently needed.

Recent research has showed a daedal association between immune cells and tumor cells for tumor development [34]. Infiltrating inflammatory cells surrounding tumor cells includes leukocytes, tumor-associated macrophages, helper T-cell, cytotoxic T-cells, T-regs, and dendritic cells, among which CD3+ T cells grab most attention. CD3+ T cells refer to total T lymphocytes, including CD4+ helper T cells, CD8+ cytotoxic T cells, and CD45RO+ memory T cells [35]. TILs have been reported to be related with favorable prognosis in several tumor types [16, 18, 36, 37], including some studies that have demonstrated that a high density of TILs was related to a good outcome for GC [38, 39]. However, there is no previously reported data about the relation between on clinical relevance of TIL density and the prognostic impact in patients with SRCC. In present study, infiltrated CD3+ TILs in the tumor microenvironment were represented immune cells. As shown in our study, tumors with high levels of CD3+ immune cells had better OS; however, CD3+ TILs was not an independent prognostic marker maintained in our multivariate analysis. Questions remain whether it is necessary to classify additional subtypes of TILs, such as CD4+, CD8+, and CD45RO+ TILs. Jin Won Kim et al. suggests that it is more accurately for CD3+ cells to predict the prognosis for patients with GC than other T-cell subtypes (CD4+ or CD8+), because CD3+ TILs, which includes both CD4+ and CD8+ cells, may reveal more convincing information about the host-tumor immune response [29].

Some previous reports have shown that EBV+ GCs have particularly abundant PD-L1-positive tumor cells and TILs. Derks et al. [40] discovered that EBV+ GCs, particularly EBV+ GC with concurrent MSI, have more PD-L1 tumor and immune cell expression compared to EBV-negative, MSS GCs. Moreover, PD-L1 expressed not only in GCs with 9p24.1 amplification, which was the genes encoding PD-L1 and PD-L2, indicating that multiple mechanisms to induce PD-L1 expression were existing in EBV+ GC. This demonstrates that immune escape mechanism mediated by PD-1 may play an even broader and crucial role in EBV+GC. Furthermore, some previous study demonstrated that more TILs were present in EBV+ GC [41–43]. And patients with the lymphoepithelioma-like carcinoma (LELC) subtype had better OS and DFS than patients with the other subtypes. In light of the above findings, TILs appear to impact the interaction between host immunity and tumor, and contribute to suppress tumor progress and recurrence in EBV+ GC patients. As mentioned in TCGA, EBV+ GC seems to benefit most from immunotherapy. Nevertheless, EBV+ GC was common in the intestinal type of GC but not in the diffuse type, which includes SRCC [19]. In our study, only one SRCC case was associated with EBV. This case was also found to have PD-L1 expression, profound CD3+ TIL infiltration, and MSI GC. Additional SRCC patients need to be recruited in future studies to further explore the clinicopathologic features and prognostic value of EBV+ SRCC.

MSI tumors are known as abundant infiltration of dense immune cell and cytokine-rich microenvironment [44–46], and has long been hypothesized to stimulate the immune system [47]. The most likely explanation is that the immune cell infiltrates associated with MMR carcinomas are directed at neoantigens. It demonstrated that increasing mutational load was associated with higher response rate to anti–CTLA-4 in melanoma and anti–PD-1 in lung cancer, indicating that the recognition of mutated neoantigen plays a crucial part in the antitumor immune response [48]. A phase II clinical trial of pembrolizumab used on patients with and without MMR deficiencies tumors demonstrated that MMR-defective tumors have higher response rate to PD-1 blockade than MMR–proficient tumors [23]. Unlike colorectal cancers, in which MSI-H is associated with better prognosis, MSI status seems to have little ability to prognosticate survival in GC patients; however, the issue is still controversial. In previous studies, the MSI-H phenotype was found to either be significantly associated with favorable prognosis [49, 50] or to not have any prognostic significance [51–53]. In the present study, MMR-defective tumors had better OS when compared with MMR-proficient tumors, but the difference did not reach the statistical significance.

In conclusion, this study demonstrated that PD-L1 and PD-1 are expressed in some patients with SRCC; however, no significant differences had been showed in clinicopathological characteristics or clinical outcomes based on PD-L1, PD-1 expression. Our findings also suggest that CD3+ TIL levels in the tumor microenvironment predict a significant difference in OS for patients with SRCC. Interestingly, we detected more infiltration of CD3+ TILs in patients with PD-L1, PD-1 positive, EBV-infected, and MSI SRCC. To our best knowledge, few studies have evaluated the prognostic value of these biomarkers in SRCC patients. Our current findings support that TILs can stimulate host immune response and suppress tumor progression. Thus, TILs in SRCC may be a biomarker for predicting patient outcome. These biomarkers, including PD-1/PD-L1, EBV, TILs, and MSI status, showed more information about the state of immune system in SRCC. These histological characteristics should be considered when selecting subgroup of patients which might benefit from PD-L1 inhibition treatment.

## MATERIALS AND METHODS

### Patients and sample collection

We identified 89 patients with SRCC from over 400 patients with gastric cancer. Specimens with histology of SRCC were collected at the Nanjing Drum Tower Hospital. None of the patients had radiotherapy, chemotherapy or other medical intervention before specimen collection. Patients were excluded if they had a history of an autoimmune disease or were taking immunosuppressive drugs. This study was approved by the Ethics Committee of Nanjing Drum Tower Hospital. Written informed consent was obtained from each individual.

### Immunohistochemical staining of tumor tissues

The expression of PD-1, PD-L1, CD3, mutL homologue 1 (MLH1), mutS homologue 2 (MSH2), mutS homologue 6 (MSH6) and postmeiotic segregation increased 2 (PMS2) in tumors was evaluated via immunohistochemical analysis. Briefly, 2-μm-thick sections were deparaffinized in xylene and rehydrated in graded alcohol. Endogenous peroxidase activity was blocked by absolute methanol containing 3% hydrogen peroxide for 15 min. The sections were microwaved for 10 min with citrate buffer for antigen retrieval, and then incubated in 1 % horse serum in Tris-buffered saline for blocking nonspecific binding. After blocking, sections were incubated overnight at 4°C with Commercially available primary antibodies using the manufacturer's instructions (anti-PD-1, rabbit monoclonal, NAT105, 1:150 dilution, Cell Marque, USA; anti-PD-L1, rabbit monoclonal, SP142, 1:100 dilution, Spring Bio, USA; anti-CD3, mouse monoclonal, PS1, 1:150 dilution, Leica/Novocastra, UK; anti-MLH1, mouse monoclonal, ES05, 1:100 dilution, Leica/Novocastra, UK; anti-PSM2, rabbit monoclonal, EP51, 1:100 dilution, Epitomics, USA; anti-MSH2, mouse monoclonal, FE11, 1:100 dilution, Dako, Denmark; anti-MSH6, rabbit monoclonal, EP49, 1:150 dilution, Epitomics, USA). Samples were then incubated with secondary antibodies for 30 min at room temperature. Then signals were visualized by incubation with 3-3′-diaminobenzidine for 5 min. The slides were then counterstained with hematoxylin before mounting.

### Evaluation of immunostaining

The PD-1, PD-L1 is observable in the cytoplasm or on the membrane of the tumor cell or the TILs. The immunoreactivity of PD-1, PD-L1 was evaluated semi-quantitatively according to the percentage and intensity of positive cells. Four grades were used to differentiate the intensity of the staining: negative, weak, moderate, or strong. Specimens in which PD-1 or PD-L1 were observed in more than 1% of tumor cells or immune cells were considered PD-1 or PD-L1 positive.

CD3 was detected in the nuclei of the TILs. The distribution of CD3+ TILs was observed in the areas with the highest density of TILs first at low magnification. The amount of positive TILs was then recorded at high magnification (HPF 400×magnification). The number of CD3+ TILs was determined in 30 random high power fields in each section.

Staining for MLH1, PMS2, MSH2, and MSH6 was regarded as positive when the tumor nuclei stained positively with the same intensity as the control tissue (non-neoplastic gastric mucosa, intratumoral lymphocytes, fibroblasts). To achieve a high sensitivity for detecting MSI in GC, any case with loss or absence of nuclear immunostaining or reduced protein expression, when compared with normal tissue, was submitted for molecular analysis of microsatellite status. Nuclear negativity and a nonconvincing immunostain of the internal positive control tissue were classified as a “missing value” (nonimmunoreactivity of single antigens).

Two independent observers carried out the immunohistochemistry analysis; both observers were blinded to any prior information on clinical or pathological characteristics of the patients' samples. If there was discrepancy between the analyses performed by the two observers, these slides were reinvestigated by both investigators using a multiheaded microscope and a consensual decision was reached.

### EBV-encoded small RNA *in-situ* hybridization

EBV-encoded RNA (EBER) was detected via chromogenic *in-situ* hybridization using fluorescein-labeled oligonucleotide probes (INFORM EBER Probe, Ventana). When >20% of the tumor cells showed staining for EBER, the case was defined as EBER positive.

### Statistical analysis

Pearson's chi-square test and Fisher's exact test were used to determine the correlation between PD-1, PD-L1 expression, infiltration by CD3+TIL, MSI phenotype, and other clinicopathological characteristics. Overall survival (OS) was measured from the time of the operation to death from any cause or the last follow-up. Survival curves were obtained using the Kaplan–Meier method and compared with the log-rank test. A univariate analysis was performed to evaluate for survival differences based on gender, age, tumor stage, the expression levels of PD-L1, PD-1, infiltrating CD3+ TILs, and MSI phenotype. A multivariate analysis was performed. The hazard ratios (HR) and 95% confidence intervals (CIs) were estimated with the use of a forward and backward Cox proportional hazards model. The correlations between the expression levels of PD-1, PD-L1, CD3+ T cells infiltration, and MSI phenotype were analyzed by Pearson correlation analysis. A p-value<0.05 was considered significant. Statistical analysis was performed using the Statistical Package for the Social Sciences for Windows version 19 (SPSS Inc., Chicago, IL, USA).

## Abbreviations

GC: gastric cancer
SRCC: signet-ring cell carcinoma
PD-1: programmed cell death protein-1
PD-L1: programmed cell death ligand 1
MSI: microsatellite instability
EBV: Epstein-Barr Virus
DNA-MMR: DNA mismatch repair
OS: overall survival
TILs: tumor-infiltrating lymphocytes
TCGA: The Cancer Genome Atlas
EBV+: EBV-positive
MSS: microsatellite stable
MLH1: mutL homologue 1
MSH2: mutS homologue 2
MSH6: mutS homologue 6
PMS2: postmeiotic segregation increased 2
EBER: EBV-encoded RNA
CI: confidence intervals
HR: hazard ratios
PFS: progression-free survival
T-regs: regulatory T-cells.
